# Supplementary material for: Curcumin, an Inhibitor of p300-HAT Activity, Suppresses the Development of Hypertension-Induced Left Ventricular Hypertrophy with Preserved Ejection Fraction in Dahl Rats
Source: Nutrients. 2021 Jul 29;13(8):2608. doi: 10.3390/nu13082608 (PMC8397934; doi:10.3390/nu13082608)
Supplement: Supplementary file 1 [file nutrients-13-02608-s001.zip › nutrients-1302780-supplementary.pdf]

# Supplementary Data

## Materials and methods

### *Measurement of interstitial fibrosis*

The excised hearts were cut into 2 transverse slices at the mid-level of papillary muscles, fixed in 10% formalin, embedded in paraffin, sliced into 4-µm-thick sections, and stained with Masson trichrome. Quantitative assessments of interstitial fibrosis area were previously described [23].

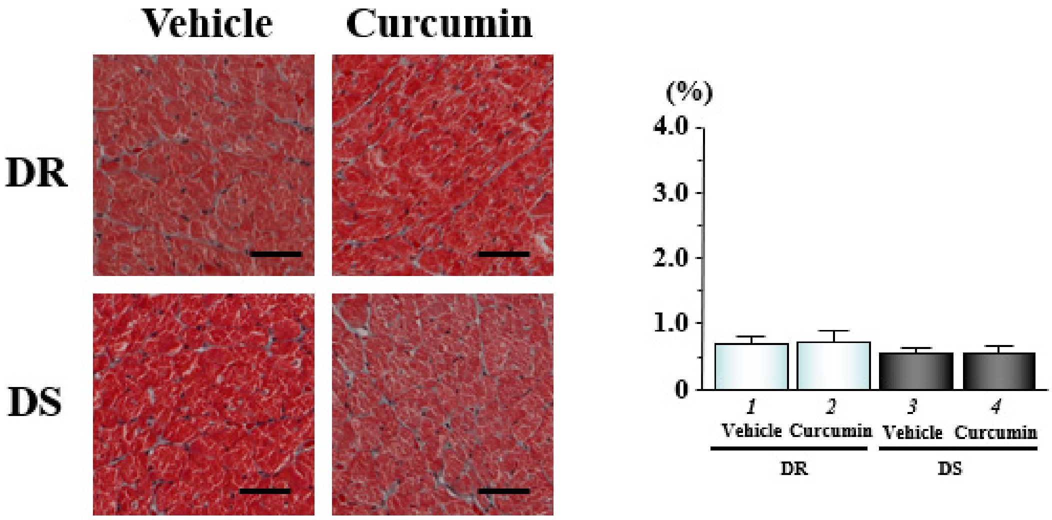


Figure S1. Curcumin treatment did not change interstitial fibrosis in DS rats. (**A**) Representative cross-sectional Masson trichrome-stained images of left ventricles in DR and DS rats with vehicle or curcumin treatment. Scale bar=50 µm. (**B**) The ratio of interstitial fibrosis area / whole image area were quantified.
